# Incidental low-grade appendiceal mucinous neoplasm in Crohn’s disease patient post ileocecal resection: a case report

**DOI:** 10.1093/jscr/rjaf294

**Published:** 2025-05-12

**Authors:** Mohammed Dakhel Aldakhil, Jawaher A Alowayyid, Mashel A Alzunidi, Raghad Ibrahim Albarrak

**Affiliations:** Department of General Surgery, College of Medicine, Qassim University, Buraydah 52346, Saudi Arabia; Department of General Surgery, Prince Sultan Military Medical City, Riyadh 12233, Saudi Arabia; Department of General Surgery, King Fahad Specialist Hospital, Dammam 32253, Saudi Arabia; Department of General Surgery, College of Medicine, Qassim University, Buraydah 52346, Saudi Arabia

**Keywords:** incidental finding, low-grade appendiceal mucinous neoplasm, appendiceal neoplasm, Crohn’s disease, inflammatory bowel disease, ileocecal resection

## Abstract

Appendiceal mucinous neoplasm is a rare tumor, found in 0.2%–0.3% of appendectomies. Inflammatory bowel disease (IBD) is a known risk factor for colorectal cancer; however, appendiceal mucinous neoplasm is rarely reported in IBD patients. Here we report a rare case in a patient with Crohn’s disease after ileocecal resection. She complained of recurrent, severe right lower quadrant pain. Further investigation revealed stenosis at the terminal ileum with a clear appendiceal orifice on magnetic resonance enterography and colonoscopy. She was referred for surgical evaluation after failure of medical management. The diagnosis of low-grade appendiceal mucinous neoplasm was confirmed by pathology after an uneventful laparoscopic ileocecal resection. Although this tumor is rare in IBD patients, a high index of suspicion is needed in those presenting with disease flare-ups, and pathological examination remains essential for diagnosis. This case underscores the diagnostic challenges and clearly highlights the importance of thorough evaluation.

## Introduction

Appendiceal mucinous neoplasm is a rare type of cancer [1]. According to World health organization WHO 2019, appendiceal mucinous neoplasm can be classified into serrated polyps, hyperplastic polyps, low-grade appendiceal mucinous neoplasms (LAMNs), high-grade appendiceal mucinous neoplasms and mucinous adenocarcinomas. [2]. Inflammatory bowel disease (IBD) is a well-known risk factor for colorectal cancer; however, appendiceal mucinous neoplasm is rarely reported in IBD patients [3]. Here we report a rare case of appendiceal mucinous neoplasm in patient with Crohn’s disease post ileocecal resection.

## Case report

A 59-year-old woman with Crohn’s disease for 30 years was switched from infliximab to vedolizumab due to bronchiectasis and pneumonia. Three months later, she developed severe colicky abdominal pain, nausea, vomiting, diarrhea, weight loss, and distension. Initial endoscopy showed gastroesophageal reflux disease (GERD), and omeprazole provided temporary relief. However, she relapsed with worsening pain, requiring multiple emergency department visits, imaging, and antibiotics, which only provided short-term relief.

**Figure 1:**
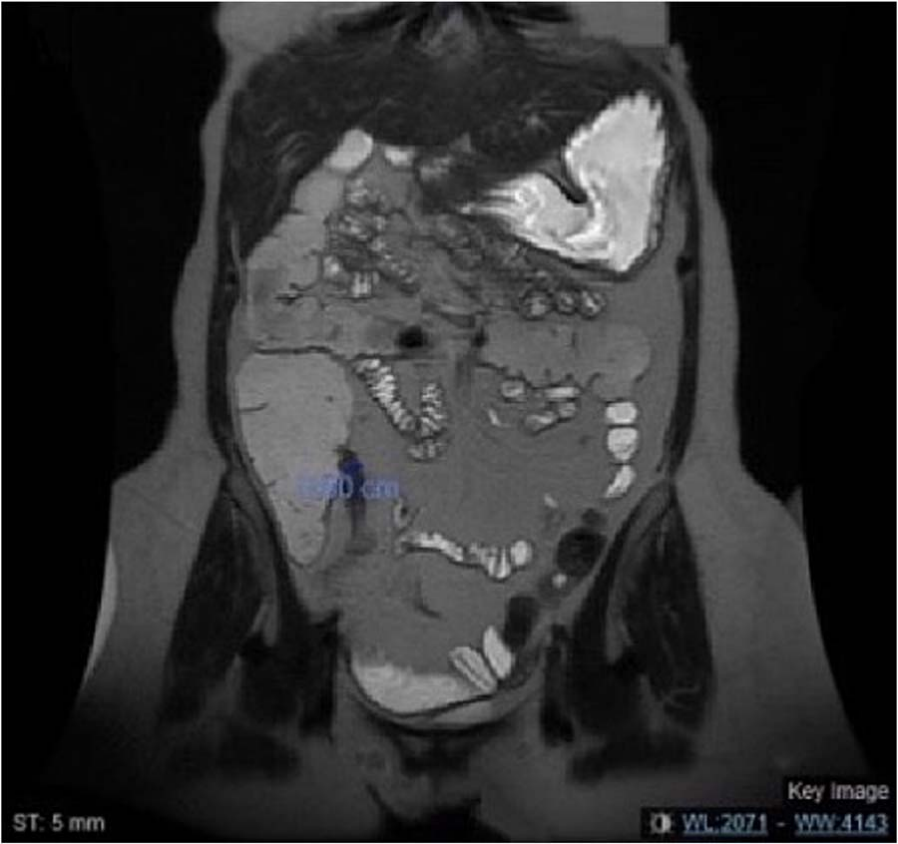
MRE of the abdomen showing a coronal view of the short segment fibrosis at the terminal ileum with mild dilated small bowel loops.

Computed tomography (CT) scans revealed progressive terminal ileal stricture with small bowel dilation and sinus tracts. Colonoscopy showed narrowing and active ileitis, leading to Budesonide treatment. Despite steroid tapering and azathioprine initiation, she had a severe flare-up with obstipation and distension. Magnetic resonance enterography (MRE) (Fig. 1) confirmed fibrosis, and she failed medical therapy after eight doses of vedolizumab.

**Figure 2:**
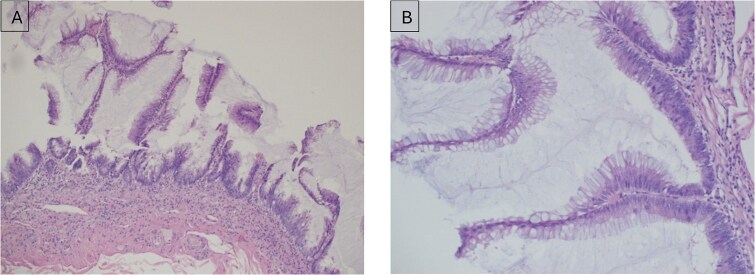
(A) Low-grade appendiceal mucinous neoplasm (LAMN) of the appendix; (B) higher magnification (× 20) of LAMN of the appendix.

She underwent laparoscopic ileocecal resection with ileocolic anastomosis, and pathology confirmed Crohn’s disease with transmural inflammation. Incidentally, a 5 cm LAMN was found with negative margins (Fig. 2). She recovered well and was discharged on postoperative Day 6 in a stable condition.

## Discussion

Appendiceal mucinous neoplasm is rare type of tumor found in 0.2%–0.3% post appendectomies [1]. The usual age of diagnosis was reported between 50 and 60 years [4]. Studies in the literature reported that appendiceal mucinous tumor is more common among female population [1, 2]. However, other literature review reported that there is no clear gender difference [2].

Appendiceal mucinous neoplasm has different clinical presentations, depending on the stage of the disease [1]. Early stages of appendiceal mucinous usually is an incidental finding post appendectomy [1, 2]. However, they could also present with right lower abdominal pain mimicking acute appendicitis, abdominal mass in the right iliac fossa, gastrointestinal perforation or intestinal obstruction [1, 5]. Late stages may present with Ascites due to accumulation of mucin, perforation, weight loss, anemia, and new onset umbilical or inguinal hernia [1]. Moreover, a cutaneous fistula, intussusception and mucus outflow from the appendiceal orifice during colonoscopy was also reported [6]. A retrospective study, showed that chronic abdominal pain was the most common presentation 37.5%, followed by acute abdominal pain in the right iliac fossa 29.2%, and 29.2% of the patients were asymptomatic at diagnosis [2]. Similarly, as in our case which clinically presented with chronic right lower quadrant abdominal pain.

Appendiceal neoplasm is difficult to diagnose radiologically as 60% of the cases was diagnosed post-operative [6]. Another retrospective study showed that 50% of the cases were diagnosed by CT scan and 37.5% was diagnosed based on the pathology result [2]. CT finings of most of the cases was cystic mass of liquid density adjacent to the caecum and the retrocecal area [2]. Moreover, previous studies showed that 67% of the patient had elevated tumor markers CEA, CA19.9 CA125, which may help in diagnosis [2]. In our case, the diagnosis was made post-operative based on pathology report.

Although IBD is a known risk factor for colorectal cancer, however, the relationship between IBD and appendiceal mucinous tumor remains uncertain [3, 7, 8]. Recent published comprehensive literature review, which included six studies, reported rare cases of mucinous appendiceal cancer in patient with IBD [9]. Appendiceal mucinous cancer was reported in 11 cases in patients diagnosed with ulcerative colitis, compared to four cases of Crohn’s disease [9].

A retrospective case control study was done on 705 patients diagnosed with IBD (either Crohn’s disease or ulcerative colitis), concluded that having synchronous colorectal dysplasia or neoplasm is associated with 15-fold increase in the prevalence of having appendiceal cystadenomas [3]. Another previous study reported that 86% of patient with IBD and colorectal carcinoma had appendiceal inflammation in the resected specimens. However, appendiceal neoplasm was rarely diagnosed [8]. Previous studies reported that obstruction of appendiceal orifice either by inflammation as in IBD or colorectal cancer may have role in developing mucinous appendiceal neoplasm [7, 10].

Treatment of appendiceal mucinous neoplasm is mainly surgical, with or without chemotherapy depending on the stage and grade of the neoplasm [1]. In LAMN surgical resection of the appendix is usually sufficient. While high grade tumors might need further management, including cytoreductive surgery, hyperthermic intraperitoneal chemotherapy, and preoperative chemotherapy trial [1].

The prognosis and the survival rate of appendiceal mucinous tumors highly depend on the stage and grade of the neoplasm [11]. LAMN treated by surgical resection has high cure rate with almost no risk of recurrence [11]. Furthermore, the 5 years survival rate for benign mucinous neoplasm is up to 100%, while the malignant type ranges from 30% to 80% [4].

## Conclusion

Although appendiceal mucinous neoplasm is a rare type of cancer in patient with IBD, high clinical of suspicions is needed in patient presenting with flare up of the disease. Moreover, appendiceal mucinous neoplasm could be easily missed radiologically; therefore, pathological examination is important in making the diagnosis. Further studies are also needed to clarify if there is a causality relationship between IBD and appendiceal mucinous neoplasm.
